# Association between clinical and IVF laboratory parameters and miscarriage after single euploid embryo transfers

**DOI:** 10.1186/s12958-021-00870-6

**Published:** 2021-12-14

**Authors:** María del Carmen Nogales, María Cruz, Silvia de Frutos, Eva María Martínez, María Gaytán, Marta Ariza, Fernando Bronet, Juan A. Garcia-Velasco

**Affiliations:** 1 IVI Madrid, Av. del Talgo, 68, 28023 Madrid, Spain; 2 Rey Juan Carlos University, Madrid, Spain

**Keywords:** Body mass index, Miscarriage, Endometrial thickness, Preimplantation genetic testing, Endometrium

## Abstract

**Background:**

The goal of this study was to investigate which factors, excluding embryo aneuploidies, are associated with miscarriage in patients who have undergone a single euploid blastocyst transfer.

**Methods:**

Retrospective, observational and multicenter study with 2832 patients undergoing preimplantational genetic testing for aneuploidies (PGT-A) due to repeated implantation failure, recurrent pregnancy loss, advanced maternal age or severe male factor were transferred one single euploid embryo.

**Results:**

One of the main findings was a significant relationship between body mass index (BMI) and miscarriage rates (13.4% in underweight women, 12.1% in normal weight, 14.5% in overweight, and 19.2% in obese women, odds ratio [OD] 1.04; 95% confidence interval [CI], 1.01–1.07 *p* = 0.006). Endometrial thickness (OD 0.65; 95%, 0.52–0.77 *p* = 0.04) and type of endometrial preparation (natural cycle or hormone replacement cycle) (OD 0.77; 95%, 0.52–0.77, *p* = 0.04) were also associated with miscarriage rates.

**Conclusions:**

BMI was strongly associated to miscarriage rates. We also observed a weaker association with endometrial thickness and with the type of endometrial preparation (natural cycle or hormone replacement cycle). None of the other studied variables (biopsy day, maternal and male age, duration of infertility, cycle length, previous miscarriages, previous live births, previous In Vitro Fertilization (IVF) cycles, endometrial pattern and/or diagnosis) were associated with miscarriage rates.

## Background

Miscarriage is a major cause of stress for couples, but also of frustration for clinicians, who cannot explain why it happens. Several studies have shown that embryo aneuploidy is the main contributing factor to failed IVF, reinforcing the relevance of PGT-A as a means of selecting chromosomally normal embryos [1–3]. The purpose of PGT-A is to select euploid embryos to transfer and improve assisted reproductive results [4].

It has been known for almost 20 years that higher body mass index (BMI) increases the odds for clinical miscarriage when compared with non-obese women, which was not observed with insulin resistance [5]. Interestingly, when analyzing the products of conception in overweight and obese women, no differences were found in the aneuploidy rates, suggesting and independent cause for the miscarriage [6, 7]. To further validate this previous hypothesis that a higher aneuploidy rate was not the cause for the higher miscarriage rates observed in high BMI patients, embryos from these women were biopsied and screened for chromosome analysis. No differences were observed in aneuploidy rate in women with high BMI versus normal weight women, suggesting that the negative impact of obesity on IVF and reproductive outcomes may not be related to aneuploidy [8]. Conversely, higher miscarriage rates were observed after euploid embryo transfers in obese women [9]. In fact, aneuploidy embryo, are not the only factor contributing to miscarriage, as evidenced by the fact that, even after PGT-A, some women still suffer pregnancy loss [10].

The aim of this study was to investigate which factors, excluding embryo aneuploidies, were associated with miscarriage rates in patients undergoing a single euploid blastocyst transfer.

## Methods

This was a multicenter retrospective study of 2832 cycles with patients undergoing PGT-A due to recurrent pregnancy loss (RPL), repeated implantation failure (RIF), advanced maternal age (AMA), and severe male factor, between 2017 and 2019 at our institution. PGT-A was only available to these indications. RPL was defined as two or more miscarriages before 20 weeks of pregnancy, RIF was defined as the absence of a gestational sac on ultrasound after the transfer of at least four good-quality embryos in a minimum of three fresh or frozen cycles to a woman under the age of 40 [11], AMA applied to women aged > 37 years, and severe male factor included azoospermia (obstructive and non-obstructive) and severe oligoasthenoteratozoospermia (sperm concentration < 1 × 10^6^ spermatozoas per milliliter, motility < 10% and morphology < 4%). Data from oocyte donation, patients with monogenic diseases and patients with an abnormal karyotype were excluded. Only euploid embryos were transferred and only the first single frozen embryo transfer per patient was included.

Mosaic embryos were also excluded from this study, since they may show lower implantation and higher miscarriage rates [12–14]. We considered mosaicism as the presence in an embryo of several cell lines with a different chromosome constitution. Therefore, embryos with chromosomally normal next generation sequencing (NGS) results in the corresponding trophectoderm (TE) biopsy were classified as euploid, embryos with chromosome abnormalities present in all the cells of the corresponding TE biopsy were classified as aneuploid, and embryos with chromosome errors observed in part of the corresponding TE biopsy were classified as mosaic. All mosaics embryos -in any percentage- were discarded from the analysis (mosaic trisomies and monosomies, segmental abnormalities in mosaic form and mosaic complex abnormalities affecting 3 or more chromosomes).

All procedures and protocols (1806-MAD-045-CN) were approved by the Institutional Review Board, which regulates and approves data-base analysis and clinical IVF procedures for research at Instittuto Valenciano de Infertilidad (IVI).

### Ovarian stimulation and oocyte retrieval

For ovarian stimulation and oocyte retrieval, patients were treated as described in previous studies [15]. Briefly, the patient received a starting dose of 150 to 300 International Units (IU) of recombinant follicle-stimulating hormone, commonly in combination with 75 IU of highly purified human menopausal gonadotropin. Gonadotropin was initiated during the first 3 days of menstruation or 5 days after discontinuation of a contraceptive pill. A daily dose of 0.25 mg (mg) Gonadotropin-releasing hormone (GnRH) antagonist was introduced when at least one follicle reached a mean diameter of 13 mm (mm). Final oocyte maturation was triggered with 0.2 mg GnRH agonist when at least three follicles reached a mean diameter of 17–18 mm. Oocyte retrieval was performed vaginally 36 h after the trigger and under ultrasound guidance.

### Embryo culture evaluation and embryo biopsy

Intracytoplasmic sperm injection (ICSI) is the technique specifically recommended for Preimplantation Genetic Test (PGT) in order to avoid the possibility, present in conventional IVF, of paternal contamination from sperm attached to the zona pellucida [16]. Maternal deoxyribonucleic acid (DNA) contamination originating from cumulus cells can also be reduced by careful denudation of the oocytes prior to ICSI [17].

Fertilization was confirmed 16–20 h after insemination by the presence of two pronuclei and extrusion of the second polar body. Normal fertilized oocytes were cultured in a microdroplet of culture medium (Life Global, IVF) until the day of the blastomere biopsy. Embryos were evaluated on day 3 and the zona pellucida was perforated using laser technology (OCTAX, Herbron, Germany) for laser-assisted hatching, followed by a trophectoderm biopsy on either Day 5 or Day 6, depending on the rate of embryo development.

We considered embryo morphology (embryo grading, quality of inner cell mass (ICM) and quality of the trophectoderm according to the classification devised by Gardner, et al. [18]

The degree of expansion was divided into the following six categories: grade 1, the blastocoel fills < 50% of the non-expanded embryo; grade 2, the blastocoel fills > 50% of the embryo; grade 3, the blastocoel fills the entire blastocyst; grade 4, an expanded blastocyst with a thin zona pellucida; grade 5, a hatching blastocyst; and grade 6, a hatched blastocyst. The ICM was graded as follows: A, tightly packed cells; B, loosely grouped cells; and C, no identifiable cells. The three TE grades were: A, many cells forming a cohesive epithelial layer; B, few uneven cells creating a loose epithelium; and C, very few large cells pushed to the side.

Embryos were classified on day 5/6 as A (high quality), B (medium quality), C (low quality) and D (poor quality). We considered high quality a hatched blastocyst, a hatching blastocyst and an expanded blastocyst with ICM (A) and TE (A). We considered normal quality a hatched blastocyst, a hatching blastocyst and an expanded blastocyst with ICM (B) and TE (B), low quality a hatched blastocyst, a hatching blastocyst and an expanded blastocyst with ICM (C) and TE (C) and poor quality a hatched blastocyst, a hatching blastocyst and an expanded blastocyst with ICM (D) and TE (D). We considered ICM (D) and TE (D) when cells were lysed.

We biopsied viable embryos with a degree of expansion that allowed us to differentiate their structures in terms of ICM and TE. In this study, ICM quality had to be at least B. We discarded 56% of the embryos because they were arrested or poor-quality embryos (embryos with ICM (D) and embryos with TE (D).

Three to five cells were removed using a laser, and samples were analyzed for next generation sequencing (NGS). Whole genome amplification with DNA barcoding was performed using the Ion Reproseq Preimplantation Genetic Screening (PGS) Kit (Thermo Fisher Scientific). Template preparation and chip loading were automated with Ion Chef. Chips were sequenced in a S5TM XL sequencer (Thermo Fisher Scientific) and the data were processed and sent to the Ion Reporter Software, version 5.4 (Thermo Fisher Scientific) for analysis. Because all trophectoderm biopsies were carried out on Day 5 or 6 of embryo development, blastocysts were then frozen using vitrification, pending on chromosomal results [19]. Chromosomal analysis was centralized in a genetic laboratory (iGenomix).

After vitrification was performed using Cryotop® (Kitazato Corporation, Shizuoka, Japan), as described in previous studies [20], all embryos were transferred in a natural or hormonally prepared cycle. Our analysis was binary and classified embryos as euploid or aneuploid. We only included data of cases in which one euploid blastocyst was transferred after warming, excluding those in which no embryos survived, approximately 5%.

In modified natural cycles, after ovarian quiescence was confirmed during menstruation, serial ultrasounds were performed from day 8–10 of the cycle. In natural cycles, once the dominant follicle reached a mean diameter of 17 mm and the endometrial thickness was ≥7 mm, 250 micrograms (μg) of recombinant human chorionic gonadotropin (rechCG) was administered, micronized vaginal progesterone 200 mg/12 h (h) was started 48 h later, and embryo transfer was scheduled 7 days after rechCG. In hormonally prepared cycles, once ovarian quiescence was confirmed using ultrasound during the first 3 days of the cycle, patients started with 2 mg/8 h oral estradiol valerate. Approximately, 10 days after initiating estradiol, if endometrial thickness was ≥7 mm and serum progesterone < 1 ng/ml, oral estrogens were maintained, micronized vaginal progesterone 400 mg/12 h was started, and embryo transfer was performed after 5 full days of progesterone administration.

### Clinical outcomes

Clinical outcomes included the implantation rate (IR), clinical pregnancy rate (CPR), clinical miscarriage rate and live birth rate (LBR). The implantation rate was calculated as the number of gestational sacs revealed on vaginal ultrasound from the 5th week of pregnancy divided by the number of transferred embryos. The clinical pregnancy rate was calculated as the number of pregnancies diagnosed by ultrasonographic visualization of one or more gestational sacs or definitive clinical signs of pregnancy divided by the number of patients undergoing embryo transfer (ET). The clinical miscarriage rate was calculated as the number of miscarriages up to the 20th week of pregnancy divided by the number of patients with positive beta Human Chorionic Gonadotropin (hCG). Clinical miscarriages were defined as those occurring after the detection of the gestational sac. The live birth rate was calculated as the number of deliveries resulting in at least one live-born baby divided by the total number of patients undergoing ET.

### Statistical analysis

Continuous variables were expressed as mean values ± standard deviations, while categorical variables were expressed as proportions (percentages), including 95% confidence intervals (CI). The ANOVA test was used for quantitative variables, whereas the chi-square test was used to compare proportions. To verify the normal distribution of the data and the homogeneity in the variances, the Kolmogorov-Smirnov test and the Leven test were applied respectively.

The odds ratio of all the miscarriage variables generated was expressed as 95% confidence intervals. A multivariate logistic regression was conducted to quantify the effect of different variables (female age, male age, body mass index, years of infertility, female etiology, male etiology, obstetric history, embryo quality, day of biopsy, modified natural cycle or replacement cycle, type of hormone replacement therapy and endometrial thickness) on the miscarriage rate.

A database was built to include all the variables included in the study classified by patient and embryo. The necessary information was exported from the clinical information manager, SIVIS (IVI database) to a table in Excel format through a database query system.

The exported data was duly encrypted to protect the clinical and personal information of the patients as provided by the applicable law in the place where the research project was carried out.

Prior to the statistical study, an exploratory data analysis was carried out to review the quality of the information extracted. Once finished, the Statistical Package for Social Sciences, version 20.0 (SPPS, IBM Corporation, NY, USA) was used for statistical analysis, and differences were considered significant if the probability of their occurrence by chance was less than 0.05.

## Results

We studied a total of 2832 cycles with patients undergoing PGT-A. Mean female age was 38.2 ± 3.5 years and mean male age was 40.1 ± 5.5 years. Mean number of oocytes retrieved was 11.9 ± 7.9, oocytes inseminated 11.1 ± 6.0, and fertilized oocytes 8.3 ± 4.7. The mean number of biopsied blastocysts and euploid blastocysts was 4.1 ± 2.8 and 2.1 ± 1.6, respectively.

The clinical pregnancy rate was 59.1%, the implantation rate was 59.1%, the clinical miscarriage rate was 13.1% and the live birth rate was 45.3%.

As shown in Table 1, we did not find differences in clinical miscarriage rates among different embryo quality groups: high quality = 11.3%; normal quality = 12.8%; low quality = 11.8%; poor quality = 12.5%, *p* = 0.83.

**Table 1:** Embryo quality and clinical outcomes

	High quality (A) (***n*** = 533)	Normal quality (B) (***n*** = 1598)	Low quality(C) (***n*** = 701)	Poor quality(D) (***n*** = 114)	***P***- value	
**Female age (years)**	**37.8 ± 0.3**	**38.3 ± 0.2**	**38.0 ± 0.3**	**38.4 ± 0.6**	**0.05**	**Not applicable** **N/A**
**Male age (years)**	**39.7 ± 0.5**	**40.1 ± 0.3**	**40.1 ± 0.5**	**40.8 ± 1.0**	**0.45**	**N/A**
**BMI (kg/m2)**	**23.0 ± 0.5**	**23.5 ± 0.3**	**23.7 ± 0.5**	**23.5 ± 1.5**	**0.24**	**N/A**
**Infertility duration (years)**	**0.8 ± 0.1**	**0.8 ± 0.1**	**0.9 ± 0.2**	**1.0 ± 0.4**	**0.87**	**N/A**
**Previous miscarriages**	**0.77 ± 0.1**	**0.81 ± 0.09**	**0.85 ± 0.2**	**0.93 ± 0.4**	**0.82**	**N/A**
**Oocytes**	**14.4 ± 0.8**^**a**^	**12.5 ± 0.4**	**11.5 ± 0.7**^**a**^	**12.2 ± 1.3**	**< 0.001**	^**a**^***p*** **= 0.02**
**Mature oocytes**	**12.8 ± 0.6**^**ab**^	**11.5 ± 0.4**	**10.7 ± 0.5**^**a**^	**10.2 ± 1.0**^**a**^	**< 0.001**	^**a**^***p*** **= 0.031** ^**b**^***p*** **= 0.027**
**Endometrial thickness (mm)**	**8.9 ± 0.2**^**a**^	**8.2 ± 0.5**^**b**^	**7.1 ± 0.1**^**ab**^	**7.5 ± 0.4**	**0.04**	^**a**^***p*** **= 0.049** ^**b**^***p*** **= 0.038**
**Implantation rate (%)**	**64.0%**^**a,b**^	**62.2%**^**c**^	**50.2%**^**b,c**^	**49.4%**^**a,c**^	**< 0.001**	^**a**^***p*** **< 0.001** ^**b**^***p*** **< 0.001** ^**c**^***p*** **< 0.001**
**Pregnancy rate (%)**	**64.0%**^**a.b**^	**62.2%**^**c**^	**50.2%**^**b,c**^	**49.4%**^**a,c**^	**< 0.001**	^**a**^***p*** **< 0.001** ^**b**^***p*** **< 0.001** ^**c**^***p*** **< 0.001**
**Miscarriage rate (%)**	**11.3%**	**12.8%**	**11.8%**	**12.5%**	**0.833**	**N/A**
**Live birth rate (%)**	**50.2%**^**a,b**^	**47.5%**^**c**^	**37.0%**^**b,c**^	**34.4%**^**a,c**^	**< 0.001**	^**a**^***p*** **< 0.001** ^**b**^***p*** **< 0.001** ^**c**^***p*** **< 0.001**

Interestingly, while the IR and CPR were influenced by embryo quality, the clinical miscarriage rate was not (Table 1).

We then performed a logistic regression analysis to investigate other laboratory and clinical variables to see if they had an impact on miscarriage rates after transferring a euploid embryo. We found that as BMI increased, miscarriage rates increased, and was significantly associated with miscarriage rates (odds ratio [OD] 1.04; 95% confidence interval [CI], 1.012–1.076 *p* = 0.006) (Table 2).

**Table 2:** Variables included in our regression model comparing clinical and IVF laboratory parameters vs miscarriage rate

Variable	OR (CI95%)	***P***-value
**Female age (years)**	0.988 (0.962–1.015)	0.98
**Male age (months)**	1.006 (0.988–1.024)	0.54
**BMI (kg/m2)**	1.044 (1.012–1.076)	**0.006**
**Infertility duration**	0.993 (0.944–1.044)	0.78
**Female etiology**	1.021 (0.971–1.073)	0.41
**Male etiology**	1.059 (0.981–1.144)	0.14
**Obstetric history**	1.008 (−0.988–1.033)	0.32
**Quality on day 5**	0.983 (0.859–1.126)	0.80
**Biopsy day**	0.986 (0.776–1.254)	0.90
**Type cycle**	0.772 (0.593–0.995)	**0.04**
**Endometrial thickness**	0.653 (0.528–0.778)	**0.04**

**Fig. 1:**
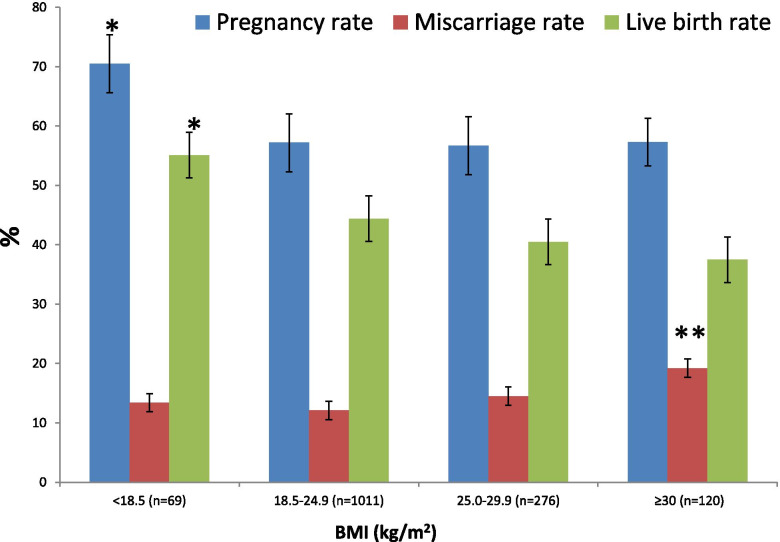
Clinical outcome in the different BMI groups. Pregnancy rate and live birth rate were significantly higher with lower BMI (*p* < 0.05) when compared to the other three groups (*). Conversely, miscarriage rate was significantly higher in the highest BMI group when compared with the other three groups (**)

We divided BMI (kilograms/meters^2^) into four groups according to World Health Organization: underweight (< 18.5; *n* = 69), normal weight (18.5–24.9; *n* = 1011), overweight (25–29.9; *n* = 276), and obese (≥30; *n* = 120). The miscarriage rate, as shown by logistic regression analysis, was significantly higher in women with obesity compared to women with normal weight, as shown in Table 3 and Fig. 1: underweight (< 18.5; 13.4%), normal weight (18.5–24.9; 12.1%), overweight (25–29.9; 14.5%), and obese (≥30; 19.2%). In Table 3, a, b, c and d indicate the statistical significance (*p* < 0.05) among groups in the post-hoc ANOVA analysis.

**Table 3:** BMI groups and clinical outcomes

	BMI < 18.5 (n = 69)	BMI 18.5–24.9 (n = 1011)	BMI 25.0–29.9 (n = 276)	BMI ≥ 30 (***n*** = 120)	P-value	
**Female age (years)**	37.6 ± 1.0	38.2 ± 0.2	38.2 ± 0.3	37.8 ± 0.7	0.36	N/A
**Male age (years)**	41.1 ± 0.6	39.9 ± 0.4	40.4 ± 0.8	39.2 ± 1.1	0.10	N/A
**Infertility duration (years)**	0.6 ± 0.3	0.7 ± 0.1	0.8 ± 0.2	1.0 ± 0.4	0.34	N/A
**Previous miscarriages**	0.6 ± 0.4	0.8 ± 0.1	0.67 ± 0.3	1.1 ± 0.5	0.27	N/A
**Oocytes**	12.6 ± 2.1	12.7 ± 0.6	12.8 ± 1.0	13.8 ± 1.5	0.37	N/A
**Mature oocytes**	11.1 ± 0.6	11.5 ± 0.4	11.5 ± 0.8	11.6 ± 1.0	0.93	N/A
**Biopsied embryos**	4.3 ± 0.8	4.5 ± 0.2	4.5 ± 0.4	4.1 ± 0.5	0.51	N/A
**Euploid embryos**	2.4 ± 0.6	2.4 ± 0.2	2.3 ± 0.1	2.3 ± 0.2	0.72	N/A
**Endometrial thickness (mm)**	8.8 ± 0.4	9.0 ± 0.2	9.1 ± 0.3	9.1 ± 0.3	0.74	N/A
**Implantation rate (%)**	70.4%^a,b,c^	57%^a^	56%^b^	56.7%^c^	0.04	^a^*p* = 0.004 ^b^*p* < 0.001 ^c^*p* < 0.004
**Pregnancy rate (%)**	70.4%^a,b,c^	57%^a^	56%^b^	56.7%^c^	< 0.001	^a^*p* < 0.001 ^b^*p* < 0.001 ^c^*p* < 0.004
**Miscarriage rate (%)**	13.4%^a^	12.1%^b^	14.5%^c^	19.2%^a,b,c^	0.01	^a^*p* < 0.001 ^b^*p* < 0.001 ^c^*p* = 0.026
**Live birth rate (%)**	55.1%^a,b,c^	44.4%^a^	40.5%^b^	37.5%^c^	< 0.001	^a^*p* < 0.001 ^b^*p* < 0.001 ^c^*p* < 0.001

When the type of endometrial preparation was analyzed natural cycle vs hormone replacement cycle, we found differences again in the miscarriage rate (9.1% vs. 13.1% respectively, *p* = 0.03 (Table 4).

**Table 4:** Type of the cycle and clinical outcomes

	Hormone replacement cycle (***n*** = 2223)	Natural cycle (***n*** = 609)	***P***-value
**Endometrial thickness (mm)**	9.1 ± 0.2	9.2 ± 0.4	0.27
**Implantation rate (%)**	59.1	58.1	0.81
**Pregnancy rate (%)**	59.1	58.1	0.12
**Miscarriage rate (%)**	13.1	9.1	**0.03**
**Live birth rate (%)**	45.2	47.4	0.36

However, no significant differences were observed in the IR (58.1% vs. 59.1%, *p* = 0.81), clinical pregnancy rate (58.1% vs. 59.1%., *p* = 0.12) and LBR (47.4% vs.45.2%, *p* = 0.36).

Additionally, and considering that we only included patients with endometrial thickness ≥ 7 mm, we observed a weak but significant association between endometrial thickness and miscarriage rates (OD 0.65; 95%, 0.528–0.778 *p* = 0.04) and when endometrial thickness was analyzed per quartiles, we did observe better clinical results with thicker endometrium (Table 5).

**Table 5:** Endometrial thickness groups and clinical outcomes

	Endometrial thickness < 7.8 mm (*n* = 686)	Endometrial thickness 7.8–8.8 mm (*n* = 734)	Endometrial thickness 8.9–10.0 mm (*n* = 792)	Endometrial thickness 10.1–12.0 mm (*n* = 488)	Endometrial thickness > 12 mm (*n* = 132)	*P*-value
Endometrial thickness (mm)	7.1 ± 0.1	8.2 ± 0.1	9.6 ± 0.2	11.1 ± 0.3	13.1 ± 0.4	0.110
Implantation rate (%)	57.0%^a^	57.4%^a^	63.7%^a,b^	62.5%^a,b^	61.8%	^a^*p* = 0.03 ^b^*p* < 0.04
Pregnancy rate (%)	57.0%^a^	57.4%^a^	63.7%^a,b^	62.5%^a,b^	61.8%	^a^*p* = 0.03 ^b^*p* < 0.03
Miscarriage rate (%)	11.2%^a^	9.2%	10.6%^b^	10.1%^c^	7%^a,b,c^	^a^*p* = 0.03 ^b^*p* = 0.04 ^c^*p* = 0.04
Live birth rate (%)	40.4%^a^	40.3%^b^	45.1%^a,b^	46.9%^a,b^	48.1%^a,b^	^a^*p* = 0.04 ^b^*p* = 0.03 ^c^*p* = 0.02

## Discussion

We investigated the different clinical and laboratory variables that could be related to miscarriage after single euploid embryo transfers. We found that BMI was strongly associated with clinical miscarriage, with increasing rates as body weight increased. Additionally, endometrial thickness and whether the embryo transfer was done in a natural or supplemented cycle were also related to miscarriage.

Embryo implantation is a critical step for a successful IVF cycle. As we know, embryo morphology has a strong predictive value for implantation, but it is far from a perfect system [21]. Chromosomal composition of the embryo after a blastocyst biopsy may be more useful to predict the outcome of the cycle and counsel our patients. Our results are in line with previous reports [18, 22]. Although other groups did find a relationship between embryo quality and miscarriage rates [23, 24], it is interesting to note that these embryos did not undergo an embryo biopsy, which may explain their findings. While some authors consider that the ICM is the best predictor for a successful pregnancy [25, 26] others deem the trophectoderm the best indicator [23]. In our patients, we transferred the embryo with the best morphology, but only after confirming euploidy.

Once a morphologically good euploid blastocyst was transferred, we analyzed the impact of the different clinical variables. We found that women with a higher BMI experienced a higher miscarriage rate than women with a normal BMI. Previous studies have shown that women with a high BMI have lower pregnancy rates and higher miscarriage rates [27–31]. It was hypothesized that these women might have a higher number of aneuploid embryos, resulting in a higher miscarriage rate. However, our study shows that even after a euploid embryo transfer, miscarriage rates remain higher. Tremellen et al. [9] first suggested a non-chromosomal cause for obesity-related miscarriage in a small series of patients. In fact, the probability of having aneuploid embryos does not change with increasing BMI [8]. In an effort to improve the reproductive outcome in women with a higher BMI, lifestyle modifications targeting a healthier life/diet and aiming to reduce their weight were looked into. Unfortunately, they did not seem to impact IVF prognosis [32, 33]. Recently, women undergoing IVF after a history of bariatric surgery showed comparable success rates relative to unoperated women with similar BMIs [34]. Although the mechanism causing this higher miscarriage rate is not yet fully elucidated, metabolic, endocrine, inflammatory and epigenetic mechanisms may be involved [19, 35–39].

Adequate endometrial preparation is crucial for successful embryo implantation. Natural cycles and modified natural cycles are easy, patient-friendly alternatives for ovulatory patients to prepare for their embryo transfer, but they require cycle regularity. An artificial cycle with estrogens and progesterone can be used with any patient, and it allows for better control of the embryo transfer for both the patient and the team, but it needs to be sustained until week 10–12 of pregnancy. When we analyzed our data, we did not find any differences in the implantation, pregnancy and live birth rates between natural and replacement cycles; we did, however, observe a higher miscarriage rate in artificial cycles.

Previous studies with frozen non-biopsied embryo transfers have shown similar results [40]. While the reason behind remains to be fully elucidated, we may speculate that inadequate progesterone priming or differences in early trophoblast invasion could be involved. Recently, it seems that artificial cycles may increase obstetric and neonatal risks [41]. Retrospective data suggests that preeclampsia may also be slightly higher in artificial cycles when compared to natural cycles [42]. However, the optimal monitoring strategy for frozen embryo replacement still needs to be established, as it depends on patient menstrual cycle regularity, the need to control the timing, and reproductive risks to be validated prospectively.

Another clinical variable to control prior to planning an embryo transfer is endometrial thickness. Adequate endometrial thickness is crucial for efficient placentation and trophoblast invasion. Defective placentation increases the risk of obstetric complications such as fetal growth restriction, low birth weight and pregnancy-induced hypertension [43]. Although ongoing pregnancies may be achieved even in very thin endometrial lining [44] and even though not all authors agree [45–48], it is well established that live birth rates are lower when endometrial thickness is less than 7 mm. In fact, when endometrial thickness was analyzed per quartiles, we did observe better clinical results with thicker endometrium, which is in line with previous research [49].

While we studied a large cohort of patients with stringent inclusion criteria, mainly single euploid blastocyst transfers, our research has some limitations. Firstly, being a retrospective study, we may just find associations but, regardless the large sample size, validation through prospective trials is advised. Secondly, the study pools data from different centers. Even though all IVF laboratories within our group share the same protocols and procedures, minor methodological differences may exist although they could be irrelevant considering again the sample size. And thirdly, although we could control for known variable that contribute to miscarriage (i.e. metabolic disturbances, Mullerian anomalies, uterine fibroids …) we could not discard all the hypothetical variables contributing to miscarriage such as KIR-HLA discrepancies, or other causative factors as yet unknown or under research that may facilitate miscarriage. However, these most likely represent a small, non-significant fraction in the whole sample.

## Conclusions

We found that BMI was strongly associated with clinical miscarriage, with increasing rates as body weight increased; additionally, endometrial thickness and endometrial preparation should be considered prior to embryo transfer to optimize success rates and minimize the risk of miscarriage.

## Data Availability

Not applicable.

## Abbreviations

IVF: In Vitro Fertilization
BMI: Body Mass Index
PGT-A: Preimplantation Genetic Test for Aneuploidies
RPL: Recurrent Pregnancy Loss
RIF: Repeated Implantation Failure
AMA: Advanced Maternal Age
IVI: Valencian Institute of Infertility
GnRH: Gonadotropin-releasing hormone
ICSI: Intracytoplasmic sperm injection
DNA: Deoxyribonucleic Acid
ICM: Inner Cell Mass
TE: Trophectoderm
NGS: Next Generation Sequencing
IR: Implantation Rate
CPR: Clinical Pregnancy Rate
LBR: Live Birth Rate
ET: Embryo Transfer
HCG: Human Chorionic Gonadotropin
OD: Odds Ratio
CI: Confidence Interval
PGT: Preimplantation Genetic Test
SPSS: Statistical Package for the Social Science

## References

[1] Litwicka K, Mencacci C, Arrivi C, Varricchio MT, Caragia A, Minasi MG, Greco E (2018). HCG administration after endogenous LH rise negatively influences pregnancy rate in modified natural cycle for frozen-thawed-euploid-blastocyst-transfer: a pilot study. J Assist Reprod Genet.

[2] Dahdouh EM, Balayla J, García-Velasco JA (2015). Impact of blastocyst biopsy and comprehensive chromosome screening technology on preimplantation genetic screening: a systematic review of randomized controlled trials. Reprod Biomed Online..

[3] Sahin L, Bozkurt M, Sahin H, Gürel A, Yumru AE (2014). Is preimplantation genetic diagnosis the ideal embryo selection method in aneuploidy screening?. J Med Sci..

[4] Munné S, Lee A, Rosenwaks Z, Grifo J, Cohen J (1993). Diagnosis of major chromosome aneuploidies in human preimplantation embryos. Hum Reprod.

[5] Mulders AG, Laven JS, Eijkemans MJ, Hughes EG, Fauser BC (2003). Patient predictors for outcome of gonadotrophin ovulation induction in women with normogonadotrophic anovulatory infertility: a meta-analysis. Hum Reprod Update.

[6] Landres IV, Milki AA, Lathi RB (2010). Karyotype of miscarriages in relation to maternal weight. Hum Reprod.

[7] Bellver J, Martínez-Conejero JA, Labarta E (2011). Endometrial gene expression in the window of implantation is altered in obese women especially in association with polycystic ovary syndrome. Fertil Steril.

[8] Goldman KN, Hodes-Wertz B, McCulloh DH, Flom JD, Grifo JA (2015). Association of body mass index with embryonic aneuploidy. Fertil Steril.

[9] Tremellen K, Pearce K, Zander-Fox D (2017). Increased miscarriage of euploid pregnancies in obese women undergoing cryopreserved embryo transfer. Reprod BioMed Online.

[10] Wang A, Kort J, Westphal L (2019). Miscarriage history association with euploid embryo transfer outcomes. Reprod BioMed Online.

[11] Coughlan C, Ledger W, Wang Q, Liu F, Demirol A, Gurgan T (2014). Recurrent implantation failure: definition and management. Reprod BioMed Online.

[12] Abhari S, Kawwass JF (2021). Pregnancy and Neonatal Outcomes after Transfer of Mosaic Embryos: A Review. J Clin Med.

[13] Munné S, Spinella F, Grifo J, Zhang J, Beltran MP, Fragouli E, Fiorentino F (2020). Clinical outcomes after the transfer of blastocysts characterized as mosaic by high resolution next generation sequencing- further insights. Eur J Med Genet..

[14] Friedenthal J, Maxwell SM, Tiegs AW, Besser AG, McCaffrey C, Munné S, Noyes N, Grifo JA (2020). Clinical error rates of next generation sequencing and array comparative genomic hybridization with single thawed euploid embryo transfer. Eur J Med Genet.

[15] Garcia-Velasco JA, Bermejo A, Ruiz F, Martinez-Salazar J, Requena A, Pellicer A (2011). Cycle scheduling with oral contraceptive pills in the GnRH antagonist protocol vs the long protocol: a randomized, controlled trial. Fertil Steril.

[16] Kokkali G, Coticchio G, Bronet F, Celebi C, Cimadomo D, Goossens V, Liss J, Nunes S, Sfontouris I, Vermeulen N, et al. ESHRE PGT Consortium and SIG-Embryology Biopsy Working Group, et al 2020 ESHRE PGT Consortium and SIG Embryology good practice recommendations for polar body and embryo biopsy for PGT. Hum Reprod Open. 2020;1–12. Available from: https://academic.oup.com/hropen/article/2020/3/hoaa020/5848312. 10.1093/hropen/hoaa020 PMC7257009 32500104

[17] Wilton L, Thornhill A, Traeger-Synodinos J, Sermon KD, Harper JC (2009). The causes of misdiagnosis and adverse outcomes in PGD. Hum Reprod.

[18] Gardner DK, Lane M, Schlenker T, Schoolcraft WB, Stevens J (2000). Blastocyst score affects implantation and pregnancy outcome: towards a single blastocyst transfer. Fertil Steril.

[19] Giles J, Meseguer M, Mercader A, Rubio C, Alegre A, Vidal C (2020). Preimplantation genetic testing for aneuploidy (PGT-A) in patients with partial X monosomy using own oocytes: is a suitable indication?. Fertil Steril.

[20] Kato K, Kobayashi T, Okuno T, Uchiyama K, Ueno S, Yabuuchi A (2014). Women’s age and embryo developmental speed accurately predict clinical pregnancy after single vitrified-warmed blastocyst transfer. Reprod BioMed Online.

[21] Munné S, Kaplan B, Frattarelli JL, Child T, Nakhuda G, Shamma FN, Silverberg K, Kalista T, Handyside AH, Katz-Jaffe M, Wells D, Gordon T, Stock-Myer S, Willman S (2019). Preimplantation genetic testing for aneuploidy versus morphology as selection criteria for single frozen-thawed embryo transfer in good-prognosis patients: a multicenter randomized clinical trial. Fertil Steril.

[22] Lou H, Li N, Guan Y, Zhang Y, Hao D, Cui S (2021). Association between morphologic grading and implantation rate of Euploid blastocyst. J Ovarian Res.

[23] Hill MJ, Richter KS, Heitmann RJ, Graham JR, Tucker MJ, DeCherney AH, Browne PE, Levens ED (2013). Trophectoderm grade predicts outcomes of single-blastocyst transfers. Fertil Steril.

[24] Van den Abbeel E, Balaban B, Ziebe S, Lundin K, Cuesta MJ, Klein BM, Helmgaard L, Arce JC (2013). Association between blastocyst morphology and outcome of single-blastocyst transfer. Reprod BioMed Online.

[25] Irani M, Reichman D, Robles A, Melnick A, Davis O, Zaninovic N, Xu K, Rosenwaks Z (2017). Morphologic grading of euploid blastocysts influences implantation and ongoing pregnancy rates. Fertil Steril.

[26] Nazem TG, Sekhon L, Lee JA, Overbey J, Pan S, Duke M, Briton-Jones C, Whitehouse M, Copperman AB, Stein DE (2019). The correlation between morphology and implantation of euploid human blastocysts. Reprod BioMed Online.

[27] Cozzolino M, García-Velasco JA, Meseguer M, Pellicer A, Bellver J. Female obesity increases the risk of miscarriage of euploid embryos. Fertil Steril. 2020; [In press]. 10.1016/j.fertnstert.2020.09.139 33267960

[28] Mahutte N, Kamga-Ngande C, Sharma A, Sylvestre C (2018). Obesity and reproduction. J Obstet Gynaecol Can..

[29] Nelson SM, Matthews P, Poston L (2010). Maternal metabolism and obesity: modifiable determinants of pregnancy outcome. Hum Reprod Update.

[30] Boots C, Stephenson MD (2011). Does obesity increase the risk of miscarriage in spontaneous conception: a systematic review. Semin Reprod Med.

[31] Supramaniam PR, Mittal M, McVeigh E, Lim LN (2018). The correlation between raised body mass index and assisted reproductive treatment outcomes: a systematic review and meta-analysis of the evidence. Reprod Health.

[32] Mutsaerts MA, van Oers AM, Groen H, Burggraaff JM, Kuchenbecker WK, Perquin DA, Koks CA, van Golde R, Kaaijk EM, Schierbeek JM, Oosterhuis GJ, Broekmans FJ, Bemelmans WJ, Lambalk CB, Verberg MF, van der Veen F, Klijn NF, Mercelina PE, van Kasteren YM, Nap AW, Brinkhuis EA, Vogel NE, Mulder RJ, Gondrie ET, de Bruin JP, Sikkema JM, de Greef MH, ter Bogt NC, Land JA, Mol BW, Hoek A (2016). Randomized trial of a lifestyle program in obese infertile women. N Engl J Med.

[33] Einarsson S, Bergh C, Kluge L, Thurin-Kjellberg A (2019). No effect of weight intervention on perinatal outcomes in obese women scheduled for in vitro fertilization treatment. Acta Obstet Gynecol Scand.

[34] Grzegorczyk-Martin V, Fréour T, De Bantel FA, Bonnet E, Merzouk M, Roset J, Roger V, Cédrin-Durnerin I, Wainer R, Avril C, Landais P (2020). IVF outcomes in patients with a history of bariatric surgery: a multicenter retrospective cohort study. Hum Reprod.

[35] Bellver J, Mariani G (2019). Impact of parental over- and underweight on the health of offspring. Fertil Steril.

[36] WHO. Obesity: preventing and managing the global epidemic: report of a WHO consultation. Geneva, Switzerland: World Health Organization;2000 (WHO Technical Report Series, No. 894). Available from: http://www.who.int/nutrition/publications/obesity/WHO_TRS_894/en. Accessed 3 June 2020. 11234459

[37] Garcia-Velasco JA, Domingo J, Cobo A, Martinez M, Carmona L, Pellicer A (2013). Five years' experience using oocyte vitrification to preserve fertility for medical and nonmedical indications. Fertil Steril.

[38] Provost MP, Acharya KS, Acharya CR, Yeh JS, Steward RG, Eaton JL (2016). Pregnancy outcomes decline with increasing recipient body mass index: an analysis of 22,317 fresh donor/recipient cycles from the 2008-2010 Society for Assisted Reproductive Technology Clinic Outcome Reporting System registry. Fertil Steril.

[39] Howell KR, Powell TL (2017). Effects of maternal obesity on placental function and fetal development. Reproduction..

[40] Cerrillo M, Herrero L, Guillén A, Mayoral M, García-Velasco JA (2017). Impact of endometrial preparation protocols for frozen embryo transfer on live birth rates. Rambam Maimonides Med J.

[41] Zaat TR, Brink AJ, de Bruin JP, Goddijn M, Broekmans FJM, Cohlen BJ, Macklon NS, van Wely M, Groenewoud ER, Mol F, ANTARCTICA trial study group (2021). Increased obstetric and neonatal risks in artificial cycles for frozen embryo transfers?. Reprod BioMed Online.

[42] Wang Z, Liu H, Song H, Li X, Jiang J, Sheng Y, Shi Y (2020). Increased risk of pre-eclampsia after frozen-thawed embryo transfer in programming cycles. Front Med.

[43] Mecacci F, Avagliano L, Lisi F, Clemenza S, Serena C, Vannuccini S, Rambaldi MP, Simeone S, Ottanelli S, Petraglia F. Fetal growth restriction: does an integrated maternal hemodynamic-placental model fit better? Reprod Sci ;2020.Available from: 10.1007/s43032-020-00393-2. 10.1007/s43032-020-00393-2 PMC8346440 33211274

[44] Cruz F, Bellver J (2014). Live birth after embryo transfer in an unresponsive thin endometrium. Gynecol Endocrinol.

[45] Wu Y, Gao X, Lu X (2014). Endometrial thickness affects the outcome of in vitro fertilization and embryo transfer in normal responders after GnRH antagonist administration. Reprod Biol Endocrinol.

[46] Traub ML, Van Arsdale A, Pal L (2009). Endometrial thickness, Caucasian ethnicity, and age predict clinical pregnancy following fresh blastocyst embryo transfer: a retrospective cohort. Reprod Biol Endocrinol.

[47] Al-Ghamdi A, Coskun S, Al-Hassan S (2008). The correlation between endometrial thickness and outcome of in vitro fertilization and embryo transfer (IVF-ET) outcome. Reprod Biol Endocrinol.

[48] Arce H, Velilla E, Lopez-Teijon M (2016). Association between endometrial thickness in oocyte donation cycles and pregnancy success rates. Reprod Fertil Dev.

[49] Ribeiro VC, Santos-Ribeiro S, De Munck N, Drakopoulos P, Polyzos NP, Schutyser V, Verheyen G, Tournaye H, Blockeel C (2018). Should we continue to measure endometrial thickness in modern-day medicine? The effect on live birth rates and birth weight. Reprod BioMed Online.

